# Isoflurane does not cause neuroapoptosis but reduces astroglial processes in young adult mice

**DOI:** 10.1186/2045-9912-1-27

**Published:** 2011-11-03

**Authors:** Renee M Dallasen, James D Bowman, Yan Xu

**Affiliations:** 1 Department of Anesthesiology, University of Pittsburgh School of Medicine, Pittsburgh, PA 15260, USA; 2 Department of Pharmacology & Chemical Biology, University of Pittsburgh School of Medicine, Pittsburgh, PA 15260, USA; 3 Department of Structural Biology, University of Pittsburgh School of Medicine, Pittsburgh, PA 15260, USA

## Abstract

**Background:**

Isoflurane, a volatile anesthetic widely used clinically, has been implicated to be both neuroprotective and neurotoxic. The claim about isoflurane causing neural apoptosis remains controversial. In this study, we investigated the effects of isoflurane exposures on apoptotic and anti-apoptotic signals, cell proliferation and neurogenesis, and astroglial processes in young adult mouse brains.

**Methods:**

Sixty 6-week-old mice were randomly assigned to four anesthetic concentration groups (0 as control and 0.6%, 1.3%, and 2%) with four recovery times (2 h and 1, 6, and 14 d) after 2-h isoflurane exposure. Immunohistochemistry measurements of activated caspase-3 and Bcl-xl for apoptotic and anti-apoptotic signals, respectively, glial fibrillary acidic protein (GFAP) and vimentin for reactive astrocytosis, doublecortin (Dcx) for neurogenesis, and BrdU for cell proliferation were performed.

**Results:**

Contrary to the previous conclusion derived from studies with neonatal rodents, we found no evidence of isoflurane-induced apoptosis in the adult mouse brain. Neurogenesis in the subgranule zone of the dentate gyrus was not affected by isoflurane. However, there is a tendency of reduced cell proliferation after 2% isoflurane exposure. VIM and GFAP staining showed that isoflurane exposure caused a delayed reduction of astroglial processes in the hippocampus and dentate gyrus.

**Conclusion:**

Two-hour exposure to isoflurane did not cause neuroapoptosis in adult brains. The delayed reduction in astroglial processes after isoflurane exposure may explain why some volatile anesthetics can confer neuroprotection after experimental stroke because reduced glial scarring facilitates better long-term neuronal recoveries.

## Introduction

Volatile anesthetics have been implicated to be both neuroprotective and neurotoxic. Several studies have shown that volatile anesthetics can reduce neurological damage from ischemia by reducing glutamate release, blocking glutamate receptors, enhancing hyperpolarization by GABA, increasing anti-apoptotic bcl-2 levels, activating p38 mitogen-activated protein kinases, and inducing neurogenesis [1-3]. Conversely, isoflurane has also been implicated in post-anesthesia cognitive decline, possible interaction and exacerbation of the accumulation of amyloid-β plaques found in Alzheimer's disease, and apoptosis in the developing brain [4,5].

It has been proposed that a prolonged exposure to volatile anesthetics is intrinsically neurotoxic and the observed protective effects are due to preconditioning, in which endogenous neuroprotective mechanisms are initiated by a limited exposure [6]. Further investigations in cell cultures suggest that isoflurane preconditioning can protect astrocytes [7]. However, other studies have showed that isoflurane impairs immature astroglia development [8]. The anesthetic effects on astrocytes remain controversial.

To investigate the mechanisms of isoflurane-induced neurological changes, degrees of neurogenesis, apoptosis, and reactive astrocytosis were studied in adult mice after exposure to various concentrations of isoflurane and recovery for different lengths of time after exposure. Similar to the results in a recent study [3], we found no evidence of brain cell death or neurogenesis in young adult mice after isoflurane anesthesia. However, our study additionally investigated the effect of isoflurane on astroglial processes. A decrease in glial fibrillary acidic protein (GFAP) and vimentin (VIM) in the hippocampal molecular layer (stratum lacunosum moleculare, SLM), dentate gyrus granular layer (DG), and dentate gyrus hilus was seen at later time points in isoflurane-anesthetized mice.

## Materials and methods

### Animal Groups

Animal protocol was approved by the Institutional Animal Care and Use Committee at the University of Pittsburgh. Sixty 6-week-old male CD-1 mice weighing 35 ± 5 g were used for the experiments. We chose to use six-week-old mice because synaptogenesis has completed in these animals [9]. The use of young adult mice also avoids the potential complications in older animals due to the recently reported anesthetic-induced changes in the neurodegenerative disorder-associated proteins [10,11]. All animals were obtained from Harlan Laboratories (Frederick, MD) and randomly assigned to different groups. Twelve mice did not expose to isoflurane and were used as controls. Forty-eight mice were assigned to three groups with continuous exposure to isoflurane at concentrations of 0.6% (n = 12), 1.3% (n = 24), or 2% (n = 12) for two hours. In order to study the anesthetic effects on cell proliferation, most animals received two intraperitoneal bromodeoxyuridine (BrdU) injections: 100 mg/kg 2 h before the exposure and 100 mg/kg after the exposure. We doubled the number of animals in the group with 1.3% isoflurane, which is close to the minimum alveolar concentration of isoflurane in humans [12], so that half of these animals (n = 12) did not receive BrdU injection to rule out any unforeseen effects from BrdU itself. The animals in the control group were handled in the same way as those in the three experimental groups except for isoflurane exposure and were randomly assigned to "recover" for 2 h (n = 4), 1 d (n = 3), 6 d (n = 2), or 14 d (n = 3) after handling. The animals in the experimental groups were also equally subdivided into the same four recovery times: 2 h and 1, 6, and 14 d after isoflurane exposure (thus, n = 3 for each subgroup in the 0.6% and 2% isoflurane groups and in the 1.3% isoflurane groups with and without BrdU injections). One mouse from each recovery time point in the control group was also randomly selected not to receive BrdU injections.

### Isoflurane Anesthesia

All mice (including the controls) were placed in a standard 9.6-liter mouse cage with a steady flow of O_2 _as the carrier gas and the desired percentage of isoflurane at a flow rate of ~2 L/min through a plastic tubing for 2 h. A calibrated Datex Capnomac Ultima Gas Analyzer (Datex Ohmeda, Helsinki, Finland) was used to ensure that the isoflurane concentration inside the cage was maintained at 0 (control), 0.6%, 1.3%, or 2%. During anesthesia, a pulse oximeter (Starr Life Sciences, Oakmont, PA) was clipped onto the hindlegs of the animals to monitor the oxygen saturation, heart rate, and respiratory rate. A temperature probe was placed under the ventral surfaces of the animals to ensure that the body temperatures were maintained at 35-37 °C. After anesthesia, mice were returned to their own cages and given 2 h, 1, 6, or 14 d to recover. To avoid any potential complications from invasive manipulations, the blood gases and end tidal CO_2 _of individual animals were not measured. However, oxygen saturation in the extremity (hindlegs) was maintained above 95%. A previous study in mice using the same anesthesia protocol and the same O_2 _flow rate showed that ventilation depression was insignificant with the isoflurane concentrations used in the present study [13].

### Tissue Preparation

After the recovery periods, mice were anesthetized with isoflurane and a transcardial perfusion was performed with buffered 10% formalin phosphate, as described previously [14-16]. The brains were extracted from the skulls and stored in buffered 10% formalin for 48 hours. The tissue was then frozen-sectioned into 30 mm slices using a Leica SM2000R (Leica, Germany) or a Lipshaw 50AB microtome. Sections from the hippocampal region were randomly selected for immunostaining as detailed below.

### Immunostaining

Standard immunohistochemistry techniques were used for GFAP, VIM, activated caspase-3, Bcl-xl, doublecortin (Dcx), and BrdU staining. Mouse IgG_1 _anti-GFAP (1:250) and Rabbit anti-Vimentin (1:200) were incubated at room temperature for 2 hours. Secondary antibodies used include: Alexa Fluor 594 donkey anti-rabbit (1:500) and Alexa Fluor 488 goat anti-mouse IgG_1 _(1:500). Rabbit anti-activated caspase-3 (1:200) and mouse anti-Bcl-xl (1:500) were incubated at 4°C overnight. Secondary antibodies used were Alexa Fluor 488 goat anti-rabbit (1:500) and Alexa Fluor 594 goat anti mouse IgG2a. Goat anti-Dcx (1:250) was incubated at 4°C overnight. The secondary antibody was Alexa Fluor 488 donkey anti-goat (1:500). BrdU staining was performed by first performing antigen retrieval by boiling tissue slices in sodium citrate buffer. Rat anti-BrdU antibody (1:200) was used as the primary antibody, and Alexa Fluor 488 donkey anti-rat (1:500) was used as the secondary antibody. Nuclear DNA was stained with 4'-6-diamino-2-phenylindole (DAPI), 1 mg/ml, in phosphate-buffered saline.

Images were acquired using Image-Pro Plus 7.0 software controlling an Olympus IX81 microscope (Olympus America, Center Valley, PA) with a Prior motorized stage, a Sutter Lambda xenon exciter light source, and an ORCA-ER digital camera. All images were tiled using Adobe Photoshop and post-processed using Image-Pro AMS (Media Cypermetics, Inc., Bethesda, MD). Doublecortin positive cell densities in the dentate gyrus subgranular zone were determined by counting all Dcx^+ ^cell soma and then normalized by the area within which the counting was performed. The percent positive areas for GFAP^+ ^and VIM^+ ^cells in the dentate gyrus granular layer, hilus, and the hippocampal SLM layer were determined by the well-established thresholding segmentation method implemented in the Image-Pro processing package. To minimize thresholding bias, all tissue sections were processed, stained, and imaged at the same time using the same batch of staining media and the same imaging acquisition setting. Two experimentalists without knowledge of the resulting positive percentage areas first determined the cut-off background values, which were then averaged for each given staining and used in the final thresholding segmentation measurements of that staining. In few cases when the background intensity of a given image was more than 1.5 standard deviations outside the averaged background intensity, the image's own background threshold was used.

### Statistics

Statistical analysis was performed using the SPSS program (IBM Co., Armonk, NY). Multivariate analyses of immunohistochemical staining were performed with respect to BrdU injection, anesthetic concentration, and days of recovery. A simple contrast test was performed between animals with and without BrdU injection, and between the control group and various groups with different isoflurane concentrations. Post hoc tests were carried out for between-group comparison using either Tukey's honestly significant difference (HSD) test, Fisher's least significant difference (LSD) test, or Dunnett t test (2-sided against the control). The results are reported as mean ± standard errors except otherwise noted. For cell counts and area-averaged cell density, comparison among different isoflurane concentration groups, including the controls, was made using the nonparametric Kruskal-Wallis test. A *p *value of <0.05 was considered significant for all tests.

## Results

**Figure 1:**
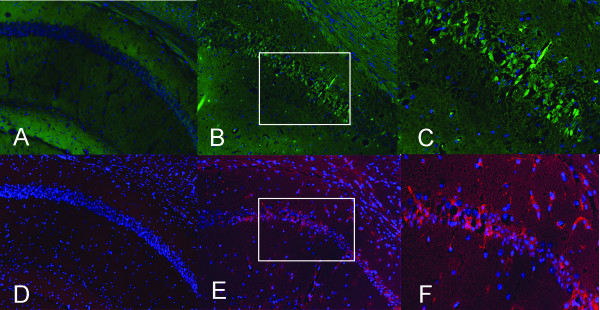
**Immunohistochemical staining of apoptotic and anti-apoptotic signals in the hippocampal Cornu Ammonis layer 1 (CA1) regions**. A-C: Activated caspase-3 staining (green) revealed no apoptosis in isoflurane-exposed animals (A), whereas positive control from a mouse after 20-min of hypoxia exhibited activated caspase-3^+ ^cells (B). C is higher magnification of area outlined in B. D-F: Bcl-xl immunostaining in the hippocampal CA1 regions (red) revealed no anti-apoptotic response in isoflurane-exposed animals (D), whereas positive control from a mouse after 20 min of hypoxia exhibited Bcl-xl^+ ^cells (E). F is a higher magnification of area outlined in E. Cell nuclei are stained with DAPI (blue).

One of the 12 mice in the 2% isoflurane group, which was pre-assigned to 6-day recovery, died unexpectedly due to unknown causes and was excluded from the analysis. To determine if 2-h exposure to clinically relevant concentrations of isoflurane can induce apoptosis in young adult mouse brains, we measured activated caspase-3 as well as the anti-apoptosis regulator Bcl-xL using immunohistochemical staining. The controls for positive staining were obtained from brain tissues of adult mice that underwent 20 min of hypoxia with 5% oxygen, which is known to cause hypoxic tissue damage. No measureable apoptotic or anti-apoptotic response was detected in the isoflurane-exposed mice, as evidenced by the absence of any positive immunoreactivity in the staining of activated caspase-3 and Bcl-xL (Figure 1). Therefore, unlike the situation in neonatal brains where apoptosis is a natural process of eliminating neurons that fail to establish synaptic connections, prolonged isoflurane exposure does not lead to apoptosis in young adult brains.

**Figure 2:**
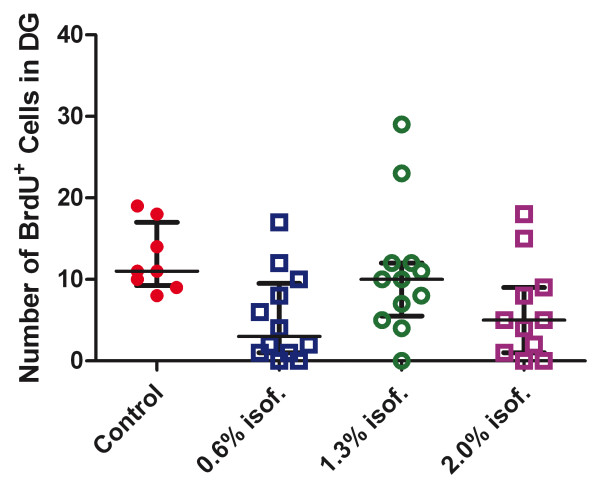
**Counting of the number of BrdU**^**+ **^**cells in the subgranular zone of dentate gyrus**. All animals that received BrdU injection were grouped by isoflurane (isof.) concentrations (Control = 0%), irrespective of recovery time. Error bars show the median and the 25^th ^and 75^th ^percentiles.

In animals that received BrdU injections before and after isoflurane exposure, we determined the isoflurane effect on cell proliferation. Figure 2 depicts BrdU^+ ^cell counts in the subgranular zone of the dentate gyrus in the four isoflurane-concentration groups (Control = 0%). When all animals with isoflurane exposure were combined as one group (irrespective of concentrations and days of recovery) and compared to the control animals as another group, the nonparametric independent-samples tests, including the Kruskal-Wallis test and median test, showed that the number of BrdU^+ ^cells in the subgranular zone of the dentate gyrus is significantly different between the isoflurane-exposed animals and the controls (p = 0.017, Kruskal-Wallis test; p = 0.029, median test). The median and the 25^th ^and 75^th ^percentiles of BrdU^+ ^cell counts in the subgranular zone were 8, 2, and 12, respectively. Seven out of eight control animals had a BrdU^+ ^cell count greater than the median, whereas 22 out of 35 isoflurane-exposed animals had a count less than or equal to the median. A closer examination of the data indicated that exposure to 0.6% and 2% of isoflurane seemed to reduce the total number of BrdU^+ ^cells in the subgranular zone of the dentate gyrus 6 and 14 d after the exposure.

**Figure 3:**
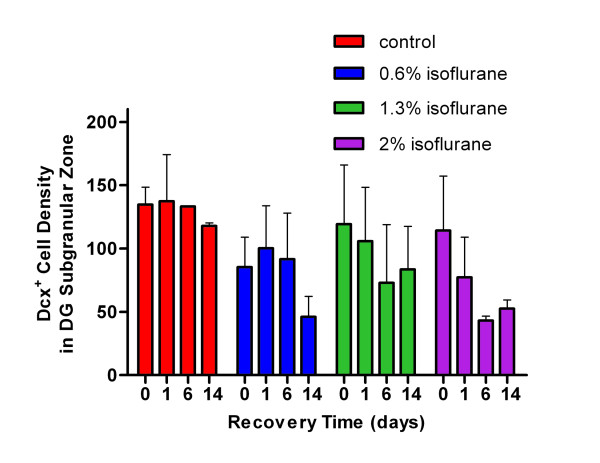
**Quantification of doublecortin**^**+ **^**(Dcx**^**+**^**) cell densities (in unit of Dcx**^**+ **^**cells/mm**^**2**^**) in the dentate gyrus subgranular zone, showing no statistically significant difference among groups**.

Neurogenesis in the dentate gyrus was measured by Dcx immunostaining. We found no differences in either the Dcx^+ ^cell counts (p = 0.055) or Dcx^+ ^cell densities (p = 0.096) in the subgranular zone of the dentate gyrus between animals in the control group and those exposed to isoflurane (Figure 3). Thus, 2-h isoflurane exposure in the 0.6-2% range had a tendency in the later recovery days to reduce cell proliferation, but did not affect neurogenesis in young adult brains.

**Figure 4:**
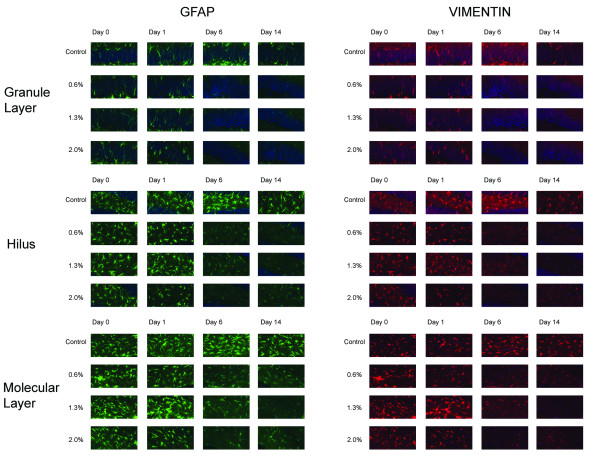
**Immunohistochemical analysis of GFAP and VIM in representative regions of the dentate gyrus granule layer, hilus, and hippocampal molecular layer**. Notice the reduced immunoreactivities at the later time points in the isoflurane-exposed animals.

To investigate the effect of isoflurane exposure on glial cells, we measured the density of glial cell processes and reactive astrocytosis in the hippocampal molecular layer and the granular layer and hilus of the dentate gyrus using GFAP and VIM staining. Astrocytes are positive for GFAP but usually become negative for VIM when they mature. However, when the brain tissue is injured or under stress, astrocytes can become reactive and re-express VIM. Microglia and radial glia are also positive for VIM. Using thresholding segmentation, we found that over the observed period, exposure to isoflurane caused a significant decrease in GFAP and VIM immunoreactivity in the three hippocampal formations studied (Figure 4).

**Figure 5:**
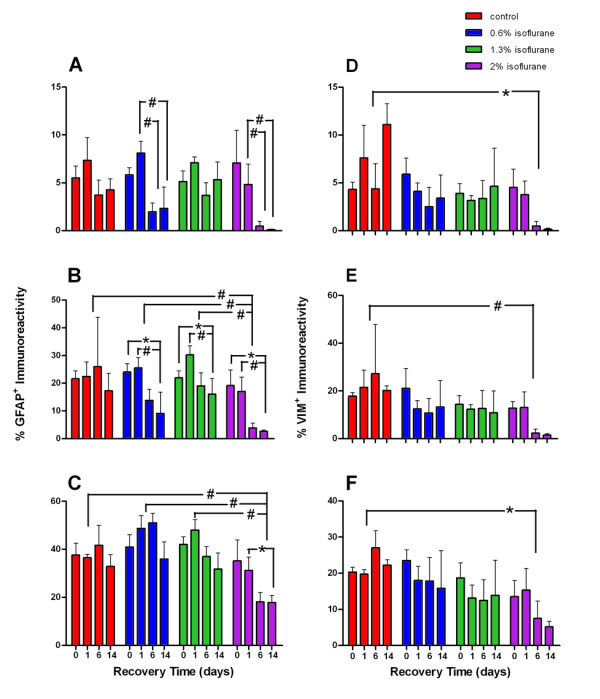
**Quantification of percent GFAP**^**+ **^**and percent VIM**^**+ **^**areas in the dentate gyrus granule layer, hilus, and hippocampal molecular layer at different anesthetic concentrations over days of observation**. Red, blue, green, and purple bars represent control, 0.6%, 1.3%, and 2% isoflurane exposure groups, respectively. Depicted are percentage of GFAP^+ ^immunoreactivity in the granule layer (A), hilus (B), and hippocampal molecular layer (C) and percentage of VIM^+ ^immunoreactivity in the granule layer (D), hilus (E), and hippocampal molecluar layer (F). Notice the marked reduction in GFAP and VIM at later time points especially at the higher concentration of isoflurane. *, p < 0.05; #, p < 0.01.

We first examined if the injection of BrdU interacted with isoflurane exposure in terms of GFAP and VIM expression. We compared the 24 animals that were exposed to 1.3% isoflurane for 2 h. Half of these animals received a BrdU injection for evaluating cell proliferation and the other half had no BrdU injection. One-way ANOVA with respect to BrdU injection showed no measureable effects on GFAP and VIM expression in the three regions analyzed (p = 0.87). We then studied the effects of anesthetic concentration and recovery time after anesthetic exposure on GFAP and VIM expression (Figure 5). Multivariate ANOVA showed that GFAP expression in SLM and in the granular cell layer and hilus of dentate gyrus were significantly affected by anesthetic concentrations and the days of recovery (p = 0.000 for anesthetic concentrations and p = 0.027 for days of recovery, with an observed power of 0.980 and 0.870, respectively, using the Pillai's Trace test). VIM expression in the three regions was significantly dependent on anesthetic concentrations (p = 0.030, Pillai's trace and Wilks' Lambda) but not on days of recovery (p = 0.202 and 0.162, Pillai's trace and Wilks' Lambda). For GFAP, post hoc pair-wise comparisons showed that GFAP expressions were significantly different in hilus between 2-h and 14-day recovery (p = 0.015) and between 1-d and 14-d recovery (p = 0.002); in dentate gyrus granular layer between 1-d and 6-d recovery (p = 0.004) and between 1-d and 14-d recovery (p = 0.007); and in SLM between 1-d and 14-d recovery (p = 0.01). Post-hoc pairwise comparisons among anesthetic concentrations showed that GFAP expressions were significantly different between the control and 2%, 0.6% and 2%, and 1.3% and 2% in hilus and SLM, but no difference in GFAP expression was found between different anesthetic concentrations in the granule layer of the dentate gyrus. For VIM, simple contrast analysis showed that 2% isoflurane exposure caused significant difference in VIM expression compared to the control in SLM (p = 0.031), granule layer (p = 0.020), and hilus (p = 0.002).

## Discussion

In this study, we investigated the effects of the volatile anesthetic isoflurane on possible mechanisms of anesthetic-induced neuroprotection and neurotoxicity in adult mouse brains. The main conclusions of this study are that isoflurane does not alter neurogenesis, nor does it cause neuronal cell death, in various formations of the hippocampus, which is known to be selectively vulnerable to insults. Instead, the clinical concentrations of isoflurane resulted in a decrease in GFAP and VIM immunoreactivity after anesthetic exposure, suggesting that glial cells and microglia may be involved in the neurological changes seen with isoflurane anesthesia.

Our results are in agreement with the recent findings of Stratmann et al [3], but differ from the conclusion derived from studies with neonatal rodents [17,18]. Although the duration of isoflurane exposure is different between the present and previous studies (e.g., 2 versus 6 h), this difference is nonessential to account for the discrepancy seen in the apoptotic responses. In developing brains, pruning of neuronal processes occurs through apoptosis to eliminate new neurons that fail to make synaptic connections. This process is essential during the synaptogenesis period. General anesthetics, particularly those that potentiate the inhibitory action of GABA_A _receptors and inhibit the excitatory action of NMDA receptors, are known to interfere with synaptic functions, including the establishment of new synapses. Thus, it is an anticipated result of the natural and protective pruning process that prolonged exposure to isoflurane will lead to the appearance of increased apoptosis in the neonatal brains undergoing synaptogenesis. The results in this study indicate that in young adult mice after the synaptogenesis period, isoflurane does not cause neuronal apoptosis. Thus, extrapolation of rodent studies to pediatric anesthesia should be approached with caution. In rodents, synaptogenesis starts to accelerate on postnatal day 7 (P7) and completes on P14 [9], whereas in humans, synaptogenesis slowly increases during the first five years after birth, accelerates to peak at age 10, and gradually plateaus around age 16 [19]. Although a direct comparison of time between humans and rodents is not possible, simply by the proportion of the anesthetic exposure time to the total synaptogenesis time, one can estimate that a 6-h continuous exposure to isoflurane [17] during the synaptogenesis period in rodents (i.e., 6 hours out of 14 days) would be equivalent time-wise to continuous administration of isoflurane for 30-65 days out of 5-10 years of synaptogenesis in humans--a clinical scenario that almost never happens. The duration of routine anesthesia procedures in children, compared to the time period of synaptogenesis in humans, is so insignificant that the amount of anesthesia-induced apoptosis, if any, is unlikely to have any long lasting impact on neuronal structuring during brain development. Further research is clearly needed to directly assess anesthetic effects on the synaptogenesis and brain development in humans.

A recent study in C57/BL6 mice found that a 2-h exposure to isoflurane induced caspase activation in a narrow time window [20]. No caspase-3 activation was detected 2 h and 24 h after isoflurane exposure, but there was an increase after 6 h. We did not detect caspase-3 activation in the current study, including at the time point of 6 h after isoflurane exposure. However, most of the animals in the current study were evaluated for caspase on Days 1, 6, and 14 after the exposure. Therefore, our conclusion is in general agreement with the previous finding. We cannot rule out the possibility that a narrow window of caspase-3 activation in CD1 mice was missed among the time points used in the current study.

Our results, however, did show some delayed effects from isoflurane exposure on glial cells, particularly astrocytes. Since astrocytes are the supporting cells of the central nervous system, it seems plausible that they could play a significant role in mediating neuronal survival versus neuronal death in response to anesthetics. Reactive astrocytosis is known to be protective early in neuronal injury as the astrocytes regulate the external chemical environment of the neurons. When the hyperplasia and hypertrophy of astrocytes are prolonged, however, injury occurs as a result of glial scarring, which creates a barrier to axonal regeneration and synaptogenesis [21]. In rats pre-treated with rat umbilical cord matrix (RUCM) cells, there was less reactive astrocytosis as measured by VIM reactivity and concurrently less neuronal loss in the hippocampus after 8-minute global cerebral ischemia, suggesting that the stem cells were able to mitigate the neuronal injury secondary to the post-ischemic reactive astrocytosis response [22,23]. Likewise, knocking down VIM expression using antisense complementary DNA for VIM [24] or preventing hypertrophy of astrocytic processes in GFAP^-/-^VIM^-/-^double knockout mice [25] can reduce glial scarring and improve neuronal regeneration and recovery after traumatic brain injuries. Therefore, the significant decrease in GFAP^+ ^and VIM^+ ^astroglia after anesthetic exposure as observed in this study may offer an alternative explanation of neuroprotection seen in other experimental studies of brain injuries involving the use of isoflurane anesthesia.

Several studies have focused on the neuronal responses to isoflurane anesthesia. In a study of traumatic brain injury (TBI) [26], CA3 neuronal survival in rats with TBI was better in those pretreated with isoflurane. The neuroprotective actions of isoflurane have been thought to be due to vasodilation and attenuation of excitotoxicity [27]. Neuroprotection by volatile anesthetics has also been observed with sevoflurane-induced neurogenesis in the dentate gyrus after cerebral ischemia. The current thought is that sevoflurane stimulates the hippocampal GABA interneurons, which in turn activate the progenitor cells in the dentate gyrus subgranular zone [28,29]. Although we did not find changes in neurogenesis after isoflurane exposure without additional insults, it is plausible that the degree of neurogenesis in the dentate gyrus after ischemia could be impacted by the amount of reactive astrocytosis; less astrocytosis after anesthetic exposure may allow for more neurogenesis as more axonal regeneration and synaptogenesis would be enabled.

In conclusion, exposure to isoflurane for two hours did not cause neural apoptosis in the adult mouse brain. Without additional insults, isoflurane exposure alone did not lead to enhanced neurogenesis in the dentate gyrus, one of the two neurogenesis regions in the adult brain. Two weeks after isoflurane exposure, the astrocytic processes, as measured by the GFAP^+ ^and VIM^+ ^areas, which are proportional to the density of GFAP^+ ^and VIM^+ ^immunoreactivity, were significantly reduced. Future studies are needed to determine if anesthetic-induced hypotrophy of glial processes will be beneficial in preventing glial scarring in the presence of other insults such as ischemia and traumatic injuries.

## Competing interests

The authors declare that they have no competing interests.

## Authors' contributions

RMD participated in the design of the study, carried out the experiments, acquired and analyzed the data, and participated in the writing of the manuscript. JDB carried out the experiments, acquired and processed the data, and participated in the data analyses. YX conceived and designed the study, secured funding for the study, provided research materials, analyzed and interpreted the data, and wrote the paper. All authors read and approved the final manuscript.
